# An Eye-Tracking-Based Investigation on the Principle of Closure in Logo Design

**DOI:** 10.16910/jemr.17.4.3

**Published:** 2024-10-10

**Authors:** Han-Yi Tseng, Hsien-Chih Chuang

**Affiliations:** Shih Hsin University, Taiwan; Chinese Culture University, Taiwan

**Keywords:** principle of closure, Gestalt psychology, eye tracking, logo design, visual communication

## Abstract

This study employs subjective evaluation and eye movement experiments to explore the application and conveyance of logo graphics design, which conforms to the Gestalt principle of closure, to understand the psychological process of this principle in the perception of a logo. The study found that there is no significant difference between completely enclosed logos and unenclosed ones that conform to the principle of closure in their influence on sightline behavior due to the effects of closure, but the subjective evaluation favors unenclosed logos as more attractive and comfortable to perceive, which agrees with modern logo design trends. In addition, the sightline distribution of the image-type logos is more scattered and has the most extended fixation duration. In contrast, the sightline distribution of text-type logos is more concentrated. Designers who understand the principle of closure can intentionally incorporate imperfection into logo design, triggering the automatic mental filling of gaps and instilling new meaning and visual effect into a design.

## Introduction

In this age of imagery, Visual Communication Design is a form of visual art that focuses on the human eye's perception. It uses symbolism to quickly and accurately convey messages, emotions, and pleasure. Its core is graphic design, which focuses on the underlying meaning through visual communication. Logo design is central to Visual Identity Design as a meaningful form of visual expression. The form of famous brand logos is deeply related to aesthetics, Gestalt psychology, and visual perception. Most people prefer structured images. The subconscious simplifies perception and actively selects more prominent visual forms as the focus of attention for mental imprinting. Logos that could be more complex and coherent often induce comprehension blockage. Barnes (11) pointed out that the design of a successful logo should be simple yet expandable across different platforms while being straightforward to perceive and easy to recall the product attributes it is trying to promote. Shape, color, and contrast are important in visual communication design. Intense light and dark contrast, or those of other colors, will cause eye saccades and attract viewer attention. According to Gestalt, These attributes can enhance visual processing and increase the effectiveness of visual communication design (73; 81).

People are generally attracted to aesthetically pleasing logos, and perhaps it is an investigation through the study of Gestalt psychology and visual perception that can be the key to unlocking this wealth of knowledge. Gestalt theory advocates the dialectical relationship between the whole and the part. It deals with the psychological mechanistic issues of visual communication design to a certain extent. It is the ideal theoretical basis for visual communication design to build a foundation in modern empirical psychology and aesthetics since it delves deeper into the basic principles of the aesthetic experience and explains visual arts from a new perspective: the relationship between visual perception and psychological experience. Designers and psychologists are constantly concerned with the issue of how the visual process and the brain work together. So, the question to be explored in this study is the relationship between logos that invoke closure, the viewing mindset, and the sense of aesthetics.

Jarodzka et al., (49) Eye tracking was developed as a way to measure the exact focal point of the eye at any given moment. It can accurately and unobtrusively measure how and where the eye is moving. Eye movement analysis technology is widely used in applied psychology research as an essential research method in psychology. This study, based on a principle of Gestalt psychology, explores the application and expressive performance of the closure principle in logo graphics design and attempts to use the quantification and subjective evaluation of eye movement psychological experiments to analyze all aspects of the viewing mindset. The purpose of this study is to understand the visual psychological process of logos that conforms to the Gestalt principle of closure, to find out the characteristics and rules of visual perception, the critical factors that affect the viewing of logos, and to understand whether there are significant differences in fixations of the eye, sightline distribution, and subjective evaluation.

Suppose designers can better understand the physiological and psychological characteristics of visual perception, effectively grasp the principles of Gestalt, and apply the knowledge to logo design. In that case, they can create good-looking logos that conform to cognitive principles, making their designs eye-catching and easy to understand and identify. Such logos can have qualities that attract the public's attention and imagination and bring aesthetic pleasure and comfort, in addition to providing new design ideas for visual communication and bringing prospects of revealing more creative artistic inspiration.

## Literature Review

### Gestalt theory

**Figure 1.**
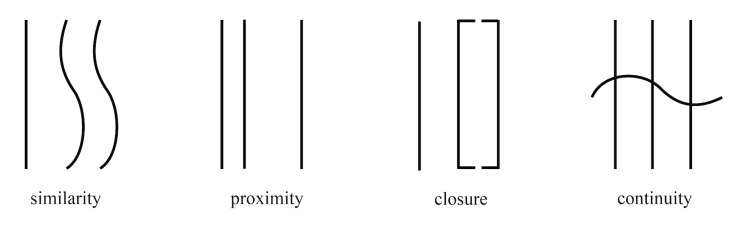
Illustration of Principles of Gestalt

Gestalt is a fundamental theory of visual design and one of the primary schools of modern Western psychology. Founded in 1912, Max Wertheimer was the first to publish a series of studies on the Gestalt principle, widely considered the beginning of Gestalt psychology. It was then advanced further by three scholars (88; 19), and studies the underlying laws of visual perception generated deep in the mind. The basic theory is that the whole is not equal to the sum of the parts (56; 92), emphasizing the process of identifying and integrating the relationship between the whole and the parts of things by the human eye (10; 63). Gestalt theory advocates the goodness of perceptual stimulation and believes that good simplified images contain conciseness and abstractness, are easy to store, extract, and process, can naturally produce pleasant feelings, and are a deterministic factor of aesthetics. It is based on holistic thinking and meaningful perception, applied to categorizing aesthetics, and blends intricately with psychological principles and aesthetics (5, 6; 38, 39; 56). Gestalt psychologists have categorized Gestalt into several principles. Some of these include proximity, similarity, continuity, and closure. If visual elements have closer proximity, are similar in shape, form a smooth contour, or are partially enclosed, this can create a grouping and effectively convey information. One such principle is the Principle of Closure, defined as the visual reduction of a complex arrangement of elements to a single recognizable graphic pattern. The brain is capable of automatically filling in the gaps between graphic elements to form a complete image (95); that is, for discontinuous and gapped images, the brain cognitively edits them to form an image that is whole and an enclosed tendency, as shown in Figure 1, the Gestalt rule is as follows (98, 99).

Gestalt psychology is widely used in visual communication design, greatly benefiting logo design, print advertising, photography, interface design, etc. Our sense of aesthetics has basic needs for wholeness and harmony. Gestalt Principles such as closure, proximity, and similarity are all related to the tendency towards what is considered 'good' graphics. Erjansola et al. (32) stated that brand logos are a fundamental part of the corporate visual identity and are a medium for carrying and transmitting information. With comprehensive analysis and abstract aesthetic design and ultimately presented as a visual graphic symbol, a logo can enhance consumers' impressions and allow for quick symbol recognition. Logo design is a process from complexity to simplification. People today prefer simple and easy-to-remember graphics since complicated and cumbersome logo graphics can quickly induce cognitive obstruction. As such, the logo design should be as simple and unique as possible, avoid appearing complicated and crowded, enhance aesthetics, and soothe the senses. The synergy of Gestalt visual principles and logo design can provide new design ideas for the designing of modern logos (90; 89). The presentation and expression of logos through visual design continue to be the explorative subject of designers (25). A good designer would be someone who fully understands the physiological and psychological characteristics of human visual perception and, with it, a grasp of what the viewer sees and wants to see. Making good use of Gestalt principles in design can bestow upon the image more profound meaning and expression, and good Gestalt design works can attract people and manipulate attention, reduce cognitive load and make it easier for people to understand and remember, generate pleasure, and be effective in the purpose of conveying the message (95).

Closure is one of the grouping principles in perceptual organization. People tend not to favor complex and loosely presented visual elements. The human eye tries to find simple and recognizable patterns, and perception connects dissociated and gapped images while the brain automatically turns them into a complete visual form. This is the basis of the principle of closure (45; 62; 41; 40).

### Closure: The intangible yet essential imaginary outline

Koffka (55) stated that a good shape is always one that is "enclosed" with edges that serve an enclosing function. Kim B. et al. (53) used neural network image classification to explore the impact of the Gestalt closure and reported that the closure effect works as a solid criterion. Closure plays an important role in contour consolidation and perceptual organization (30; 31; 29; 35; 67; 54; 58). Gregg Berryman (41) pointed out that closed shapes are more visually stable than unenclosed shapes. People naturally tend to shrink gaps and complete unfinished shapes, while open and incomplete shapes tend to scatter attention (83). Therefore, many studies concur that closure has a strong impact on perception. Closed contours are quicker and easier to perceive and distinguish than open contours and processing efficiency is higher and more accurate; they have improved recognition and higher effectiveness in capturing attention. (17; 30; 12; 67; 58; 15; 74). Humphreys and Riddoch (47) found that subjects performed better when perceiving closed shapes and were more likely to recognize presented text when they were closed.

### Application of the Principle of Closure in Logo Design

**Figure 2.**
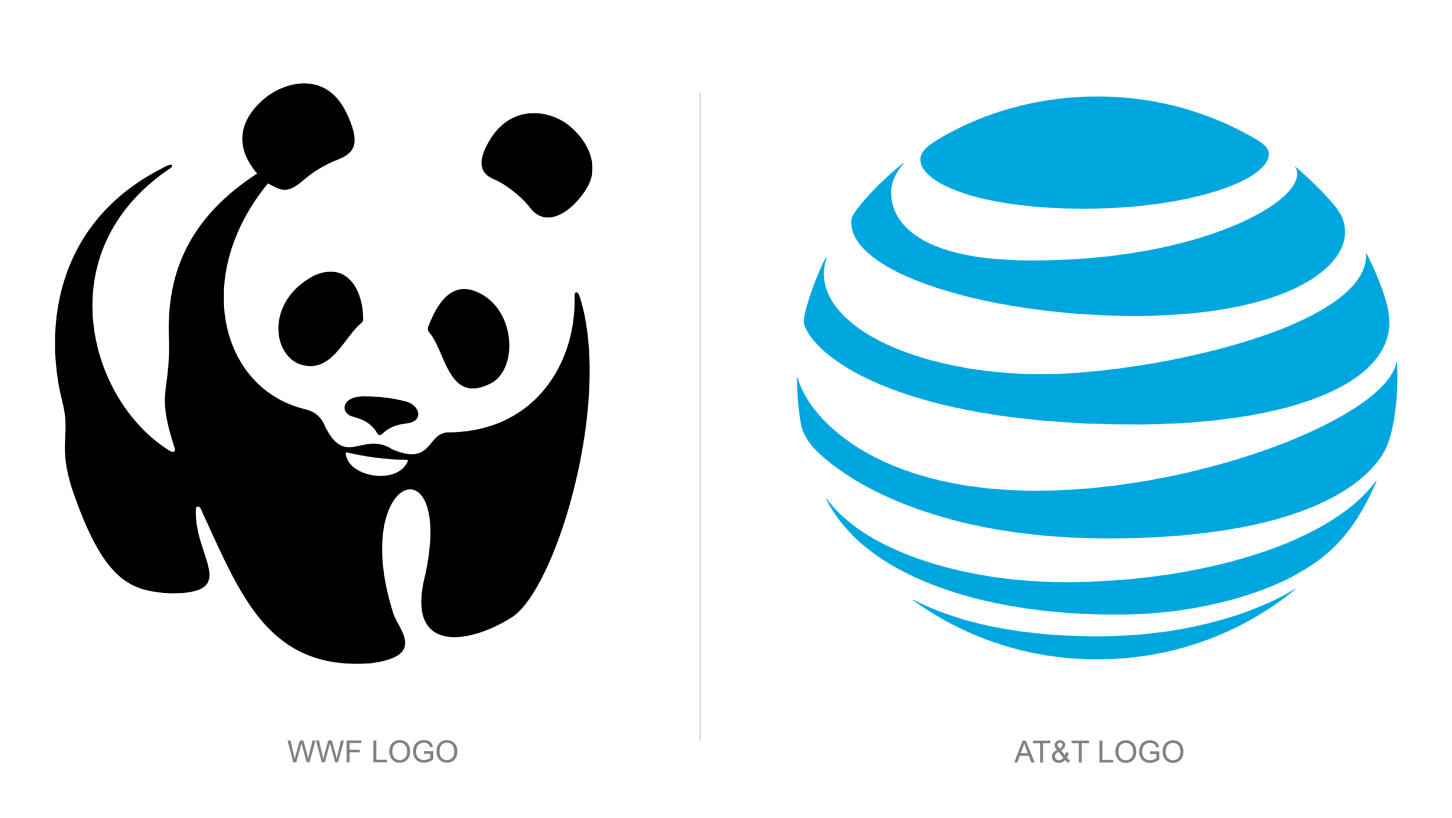
An example of a brand logos designed using Gestalt's principle of closure

**Figure 3.**
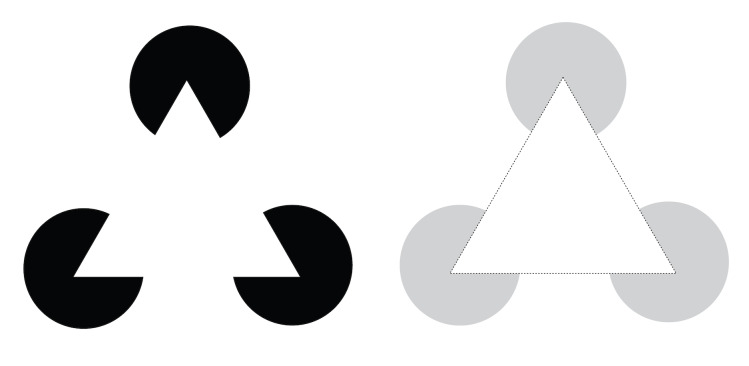
Principle of Closure-Unseen yet essential line

The principle of closure is one of the most fundamental organizing principles of Gestalt theory and is most commonly used in visual identity design (62; 52). Today, many well-known brand logos are designed using the Gestalt principle of closure (as shown in Figure 2). The least number of lines are used to achieve the most vivid visual effect, making the human eye automatically complete and enclose an image to be perceived as a whole. As shown in Figure 3, although there seem to be three circles with slices missing, when aligned as depicted, we perceive the outline that fills the gap as a complete triangle that covers three circles (99). Another example is the panda logo of the World Wildlife Fund. It can be seen that the curves of the panda are not connected. However, with closure, our brain fills in the lack of graphics to be wholly recognized and suppresses the excessive interference from fragmented shapes on vision. In this case, arcs that do not exist were innately created to aid identification (62; 52; 94).

The principle of closure applied to designing a logo can bring out visual congruency while circumventing blandness. It allows for innovation in creating a more vivid, novel, and attractive logo design that is able to enhance visual recognition with a form that is easy to propagate and attract viewer attention. (90; 95; 62; 52). The design of a logo conforming to the principle of closure exploits the psychological and cognitive tendency to make complete images. This can mitigate monotony, induce curiosity, unconsciously create a more profound impression, and stimulate the imagination. The principle of closure allows for the practical identification of logos, such as images missing specific segments that can still be recognized. Arnheim (7) once said that when the human eye sees an exceedingly simplistic image that overly emphasizes order, feelings of monotoneity and rigidity can easily result in viewing fatigue. Therefore, some complex, slightly asymmetrical, and disorganized graphics, such as incompletely enclosed (unenclosed) type graphic design, give people a simplistic, relaxed, and liberating feeling (60; 97). However, as the missing parts increase, an image's tendency to be recognized gradually decreases as the parts become insufficient for converging into an easily recognizable whole image. Therefore, designers must pay attention to and masterfully balance simplicity and complexity to achieve recognition (94).

As to the application of the principle of closure in design-related eye movement research, Xinran Hu and Dinko Bačić (46) conducted exploratory research using eye-tracking technology to understand the viewing behavior of the Gestalt principles of proximity, similarity, continuity, and closure. They found that the principle of closure exhibits better visual attention retention to competing principles, a property of Gestalt that can be effectively utilized in design. Chuang et al. (24) used eye-tracking technology to explore the impact of the Gestalt principle in photography on the viewer's visual cognitive process. It was found that Gestalt photography has a significant impact on the number of fixations, sightline distribution, and subjective evaluation of aesthetics and complexity. Closed composition photography exhibits properties of the principle of closure, and since the image is simple and easy to recognize, the number of fixations and saccades was the least, the fixation time was the longest, and the aesthetic feel was the strongest due to the centralized sightline.

These findings are consistent with the research results of Qutb M et al. (76), who used eye tracking to explore the application of Gestalt in outdoor advertising. Closed-composition images give off a sense of completeness with a tendency to simplify recognition, reduce the number of fixations, generate the longest viewing time, and produce more focused sightlines. The strong aesthetics effectively guide the viewer's sightline to create a natural visual association. Hu (45) further used eye-tracking research to explore the psychological impact of the principle of closure by analyzing gap sizes and the golden ratio. The study showed that the application of the golden ratio in visual design is correlated to visual harmony and pleasantness.

The principle of closure is most commonly used in visual identity design employed by many well-known brands in their logos. Summarizing the above, we know that the closure property is very important for the visual perception and cognition of images. Enclosed designs in either advertising or photography can exhibit the properties of the principle of closure (76; 24). In particular, enclosed images exhibit significant effects of the property of closure compared to open outline images. However, the difference in effect between complete enclosure and partial contour concealment on logo design is not yet clear. This study further explores the psychological process of viewing logos with the principle of enclosure properties and the differences in the influence on eye movement data and subjective psychological evaluation between logos that are fully enclosed and those with some concealed contours (partially enclosed).

### Eye Movement

Eye movement analysis is an essential psychological research method; it extrapolates eye movement data representing psychological activity. This is a method of glimpsing into the inner mind by looking at the position and movement process of the eyeball, and through the objective and precise analysis of eye movements, the complex visual cognitive process can be captured effectively and immediately. Observing people's eye activities can reveal many psychological activities. eye movements include various types such as fixations, pursuits, or saccades. (2; 34; 69 & 86), Key indicators include fixation duration and fixation count (9; 57). Rayner (78) pointed out that observing the sightline trail makes it possible to directly infer the cognitive process of higher brain functions. Henderson and Hollingworth (43) pointed out two crucial items in observing eye movement: the area where people are fixated when watching images and the fixation duration. Since the movement of the eyeball reflects the immediate process of watching an image, observation of this provides an understanding of the areas and positions of interest. The position of fixation and the area of attention distribution are, therefore, strongly correlated (3; 27; 43; 44; 65; 68).

This research uses an eye-tracking apparatus from the standpoint of empirical aesthetics, physiology, and psychology to explore the viewing process of enclosed property logos. Fixation density is a reliable indicator of interest in a particular segment of an image (4; 57). A topographical information system was utilized as a descriptive method for the Spatial Dispersion Index and used to reveal the fixation distribution level difference of the property of closure in images by compiling the data for the logo viewing process. Through this quantification of eye movement data, an in-depth understanding of the influence of the principle of closure on eye movement can be achieved. In addition, the subjective evaluation also reveals the psychological experience of an enclosed structure. Further compiling the data into a heat map for qualitative analysis may be constructive for designers as a reference and apply to designs that require increased recognition and aesthetics.

### Research Variables

1. Number of fixations: The number of fixations refers to the total number of times the eye is fixated on a certain point, which is the most critical primary indicator of eye movement research. It reflects the complicacy of the external stimulus at the moment of fixation and measures an individual's mental process's direction or internal focus (43). The number of fixations is related to the number of visual components an individual needs to process. Longer and more difficult comprehensive tasks usually require more fixations and longer fixation duration (37; 48; 78; 87).

2. Fixation Duration: Fixation duration refers to the time that the eyeballs appear to be temporarily stationary in a particular area when viewing, and the unit is calculated in milliseconds (ms). Fixation duration has been used to study cognition and attention. It reflects the complicacy of the external stimuli at viewing (72; 80). The longer the fixation duration, the more complex the information is to understand or the more attractive the target is to the viewer (51), Sugano et al. indicated that duration is the feature that contributes the most to estimating the user’s interests (84; 82).

3. Saccade duration: Saccade duration allows the eyes to move between two fixation points. It is closely associated with the saccade distance, and an increase in the distance means an increase in duration. The duration when the eyeball starts to jump rapidly and when it is ready to jump are two mechanisms almost distinctive in physical and psychological functioning (36). Yarbus (96) showed that saccade time is closely and positively correlated to saccade distance. The average speed of saccades is typically up to 500 degrees of visual angle per second, with sac-cades of 2 degrees of visual angle taking approximately 30 milliseconds and saccades of 5 degrees of visual angle taking nearly 50 milliseconds (1). The distance of saccades is also related to the nature of the task being performed. For example, when reading, each saccade has a distance of about 7-9 English letters, or about 2.5 to 3.3 Chinese characters (85), but when viewing pictures a saccade can be as far as four to five degrees (77). Thus, the length of the saccade reflects the density of the message, that is, the greater the density of the message, the shorter the length of the saccade (20). Therefore, the greater the complexity of the logo design and the amount of information, the longer the saccade duration will be.

4. Number of saccades: Refers to the duration the eyeball moves between fixation points (2), the velocity of which can reach 800°/s (100). Usually, the saccade between fixation points is used to measure attention shifts related to processing visual information (51; 59; 64), with fewer saccades representing more efficient searching (28), so the more complex the logo design, the more the number of saccades as comprehension becomes more challenging.

5. Spatial dispersion index (SDI) of fixation: Fixation dispersion is a novel index that uses a topographical information system to depict the distribution of fixation points and can compensate for the lack of spatial information portrayal with eye tracking. Ma and Chuang (66) used the spatial dispersion index (SDI) to analyze the differences in the distribution of fixation of different Chinese character block types. It was found that the SDI value of enclosed structure type characters had the lowest distribution, and the overall sightline of the enclosed structure is more concentrated than that of the unenclosed structure. This is used to understand the degree of dispersion of the fixation distribution of a viewer watching the image. The more concentrated the fixation points, the lower the degree of dispersion, and the smaller the SDI (24; 66; 91).

6. Heat Map: The heat map converts the recording of the entire stimuli viewing process into an intuitive visual image to reveal areas of concentration and fixation durations. Multiple recordings enhance understanding of sightline consensus and attention distribution (14)

7. Complicacy: Complicacy refers to the subjective perception and cognitive load from the complications of comprehending the logo. Complicacy is influenced by factors such as increased constituent components or excessively cluttered visual stimuli, which are difficult to distinguish. Complicacy is related to viewer perception, motivation, and aesthetics. (8; 22; 23; 70; 71; 78) people have different subjective complexity and cognitive load for different types of logos.

8. Aesthetic evaluation: The viewer's evaluation of the aesthetic qualities of the logo. Berlyne (13) found that users like certain websites generate aesthetic emotions and synaesthesia, such as interest and pleasure.

## Methodology

### Experimental design

**Figure 4.**
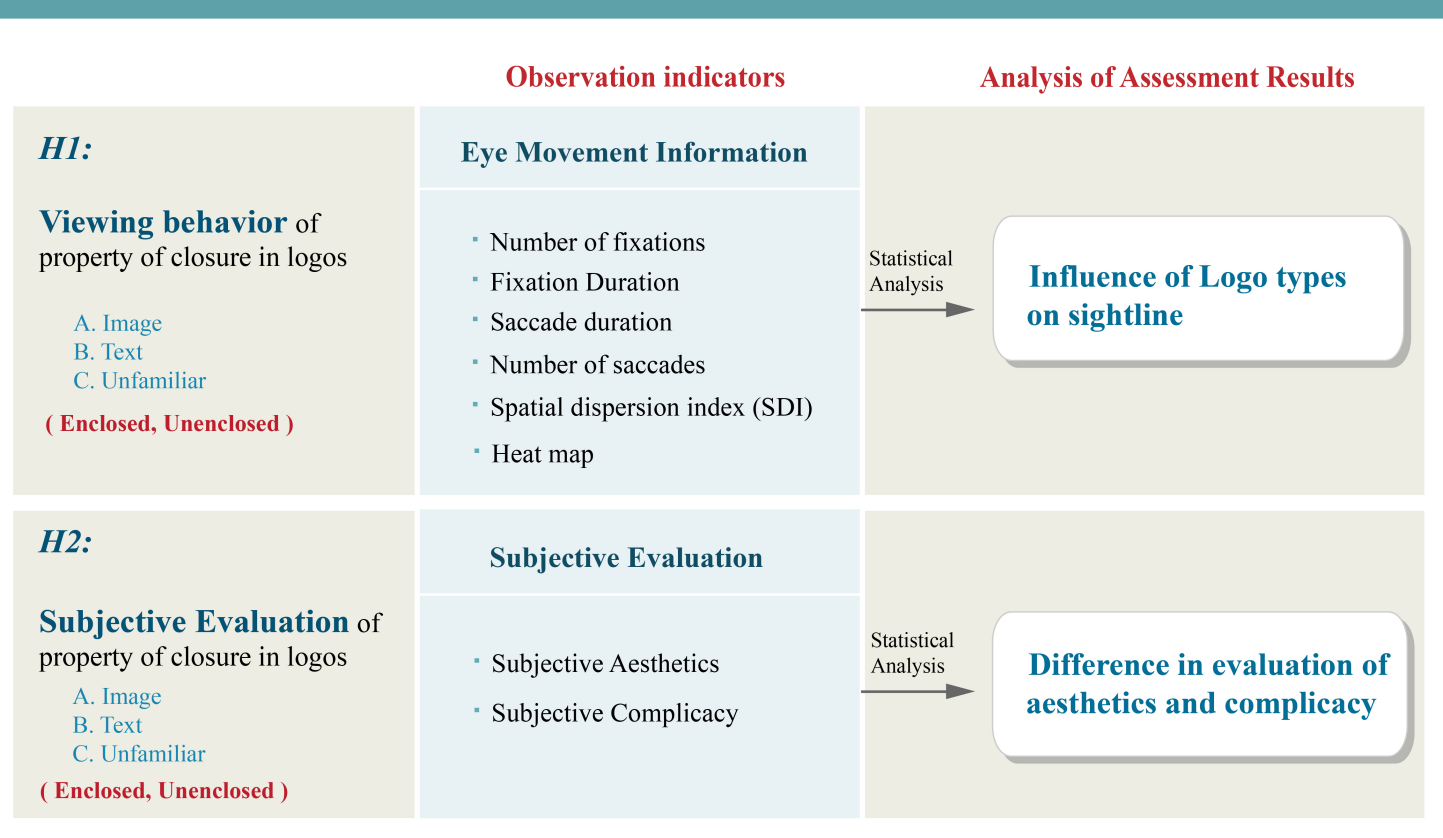
Research Structure

The primary purpose of this experiment is to explore the principle of closure and the psychological process of vision. The experiment manipulated the closure property of logos and its influence on eye movement information to investigate critical factors that affect the viewing and recognition of logos, particularly paying attention to the differences in sightline distribution and subjective evaluation between enclosed and unenclosed logos. This research can be a reference for visual design creation and art education. The research structure is shown in Figure 4.

### Participants

In this experiment, willing participants were recruited in exchange for monetary compensation. A total of 21 participants were gathered, including 11 males and 10 females between 18 and 20 years old, averaging 19 years old. Participants signed a consent form for the experiment; all had normal vision or wore corrective lenses. The experimental design randomly presented stimuli using materials of enclosed property and applied them using a within-subject design. All participants were subjected to the entirety of the experiments.

### Experimental stimuli

**Figure 5.**
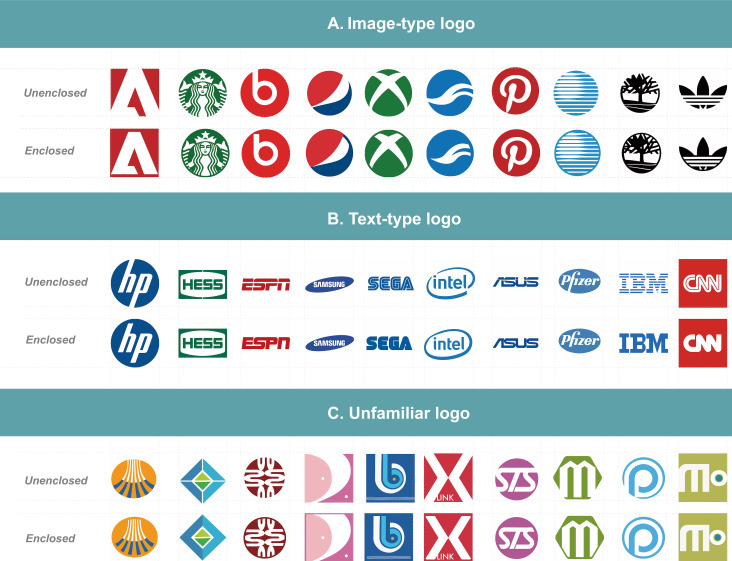
Stimulus material

The experimental pictures are selected from the well-known brand logos of Fortune Global 500 ranking companies from the American "Fortune" magazine. The experimental photos were then screened using the focus group interview method with three invited experts, each having over ten years of field experience. The experts were interviewed, and dialog was exchanged in group discussion to select photos that pertain to the Gestalt principle of closure. The experts then gave a score from 1 to 7 points on the Likert scale, with 1 point indicating very unfitting and 7 points indicating very fitting. This was performed for all enclosed and unenclosed logos as experimental samples. Finally, an additional set of experimental pictures was produced according to the principle of inductive arrangement by Gestalt psychologist Zakia (99). The previously selected logos were re-drawn as the experimental group that conforms to the closure principle into a wholly enclosed and partial group. The enclosed graphics and text logos and unenclosed graphics and text logo groups each contained ten logos for 40 pictures. In addition, to mitigate the effect of currently existing logs having imprinted on the subjects, five additional self-designed brand new and never before seen logos were added to each of the four above groups for 20 brand new pictures. This makes 60 pictures for the experiment (as shown in Figure 5). Each picture is 1000*1000 pixels in size.

The experimental manipulation is as follows:

**Independent variable:** Completely enclosed (experiment group) and partially enclosed logos (control group) within the principle of closure

**Dependent variables:**1. Eye movement information (total number of fixations, total fixation duration, total number of saccades, total saccade duration, SDI fixation spatial dispersion) 2. Subjective evaluation (subjective complicacy, aesthetics).

Hypothesis 1: There is no difference in eye movement information between a completely enclosed logo and an unenclosed logo that conforms to the closure principle, as the principle dictates that gaps would be automatically filled in.

Hypothesis 2: The logos that conform to the closure principle have a highly subjective psychological aesthetic feel and look more comfortable and pleasurable.

### Experiment Procedure

Before the formal experiment, the subjects were asked to sit about 60 cm before a 21-inch CRT screen in a standard monitoring site. The center of the screen was in a straight line with the subjects. The screen took about 36.8 degrees of the subject's field of view, and the vertical height was about 28.1 degrees above the horizontal eye line. Then, the eye tracker (Tobii Pro Nano eye tracker) was set up for the subjects at a sampling frequency of 60Hz to record the sightline trail of the right eye, and a 9-point calibration was performed to configure the eye tracker to collect data.

**Figure 6.**
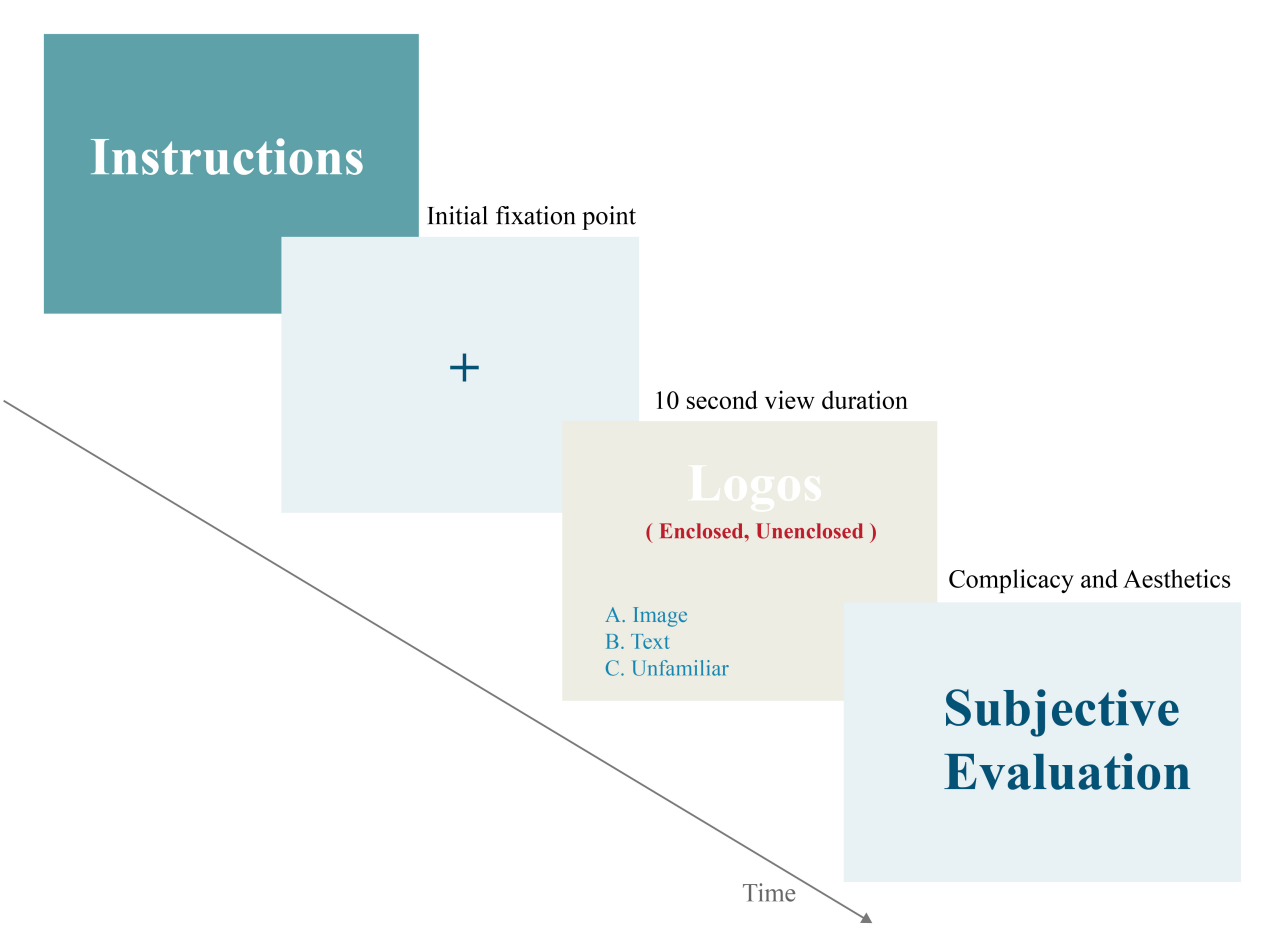
Experiment Flowchart

At the beginning of the experiment, the participants practiced to become familiar with the operation of the experiment setup using the given instructions. In the formal experiment, images of logos were presented. Under the condition of comprehensively viewing the picture, the subjects' sightline information was recorded entirely in the process. There were 60 images in the experiment, and each image was presented for 10 seconds, the order of which was randomly determined by the computer. Drift correction was performed for every ten images shown. After the images were all shown, the subjects were asked to conduct a subjective evaluation of the 60 logo images. A complicacy score of 1 to 7 points, the most straightforward scoring 1 to the most complicated scoring 7, and an aesthetics score of 1 to 7 points, 7 points being very good-looking, were given. The total experimental time lasted 15 minutes, and the experimental process is shown in Figure 6.

## Results

### The Influence of Enclosed Logo on an Eye Movement

**Figure 7.**
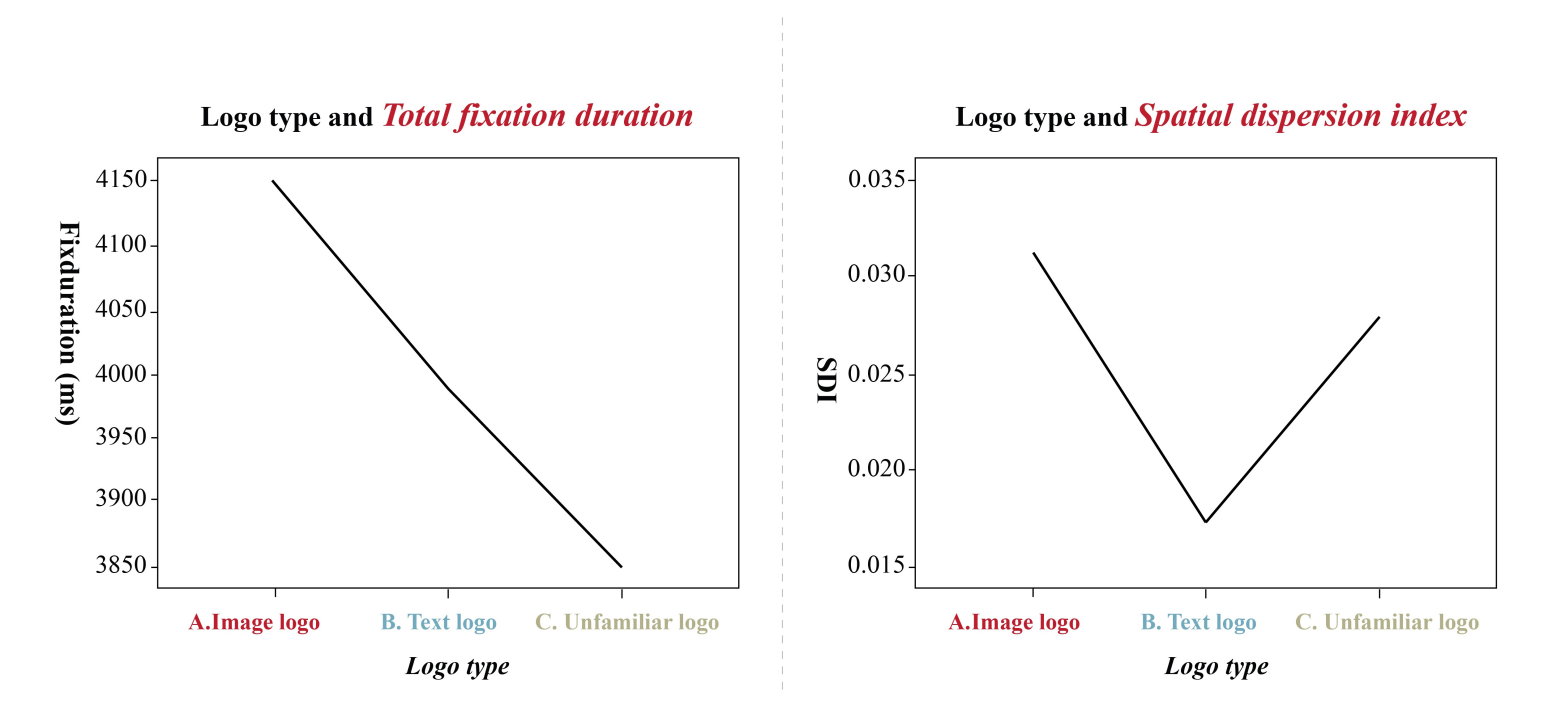
fixation duration and sightline distribution

The statistical results of this experiment were analyzed using SPSS22 statistical software. Investigation into the sightline differences between enclosed and unenclosed logos and confirmation of whether it affects eye movement information in terms of total number of fixations, total fixation duration, total saccade duration, number of saccades, and SDI variance on dependent variables was performed. Results reveal that total number of fixations (*F*(1, 20) = 0.240, *p* > 0.05), total fixation duration (*F*(1, 20) = 0.096, *p* > 0.05), total saccade duration (*F* (1, 20) =0.099, *p* > 0.05), and number of saccades (*F*(1, 20) = 0.159, *p* > 0.05) all had no significant difference between enclosed and unenclosed logos (as shown in Table 1), indicating that enclosure on the logo or not had no effect on fixation duration and number of fixations, and does not influence the overall sightline in logo recognition. Furthermore, analysis was performed on the SDI-dependent variables that reflect the breadth of the fixation point distribution in the logo structure. ANOVA analysis of variance showed that the logo conformity to the principle of closure had no significant main effect on the SDI gaze spatial dispersion dependent variable (*F*(1, 20) = 0.072, *p* > 0.05); however, different types of logos had a significant main effect on the dependent variable of SDI gaze spatial dispersion (*F*(1, 2) = 0.2315, *p* <0.01), so it appear that different types of logos will exhibit different level of distribution, and image logos have more scattered sightlines. In contrast, those of text logos are more concentrated. Different types of factors have a significant main effect (*F*(2, 40) = 7.917, *p*<0.01) on the total fixation duration dependent variable, and it appears that different types of logos have different fixation duration, and image logos experience longer fixation duration than text logos, as shown in Figure 7.

**Table 1.**
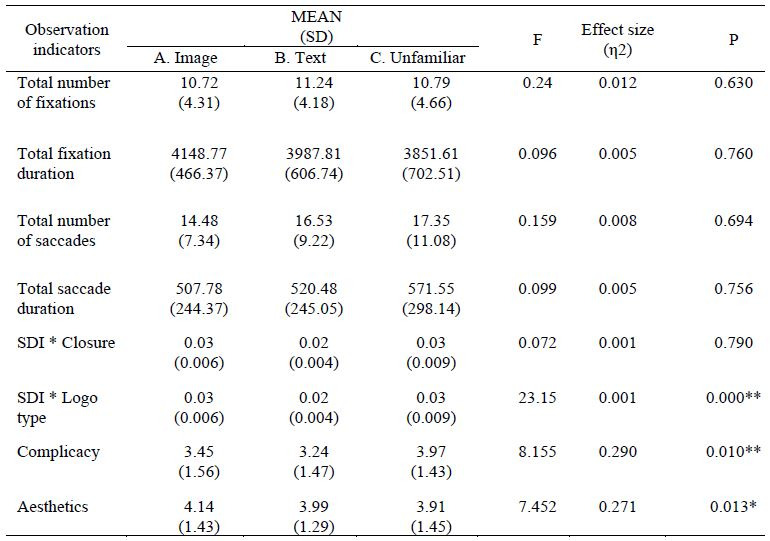
Table of ANOVA

Note. A. Image B. Text C. Unfamiliar

**Figure 8.**
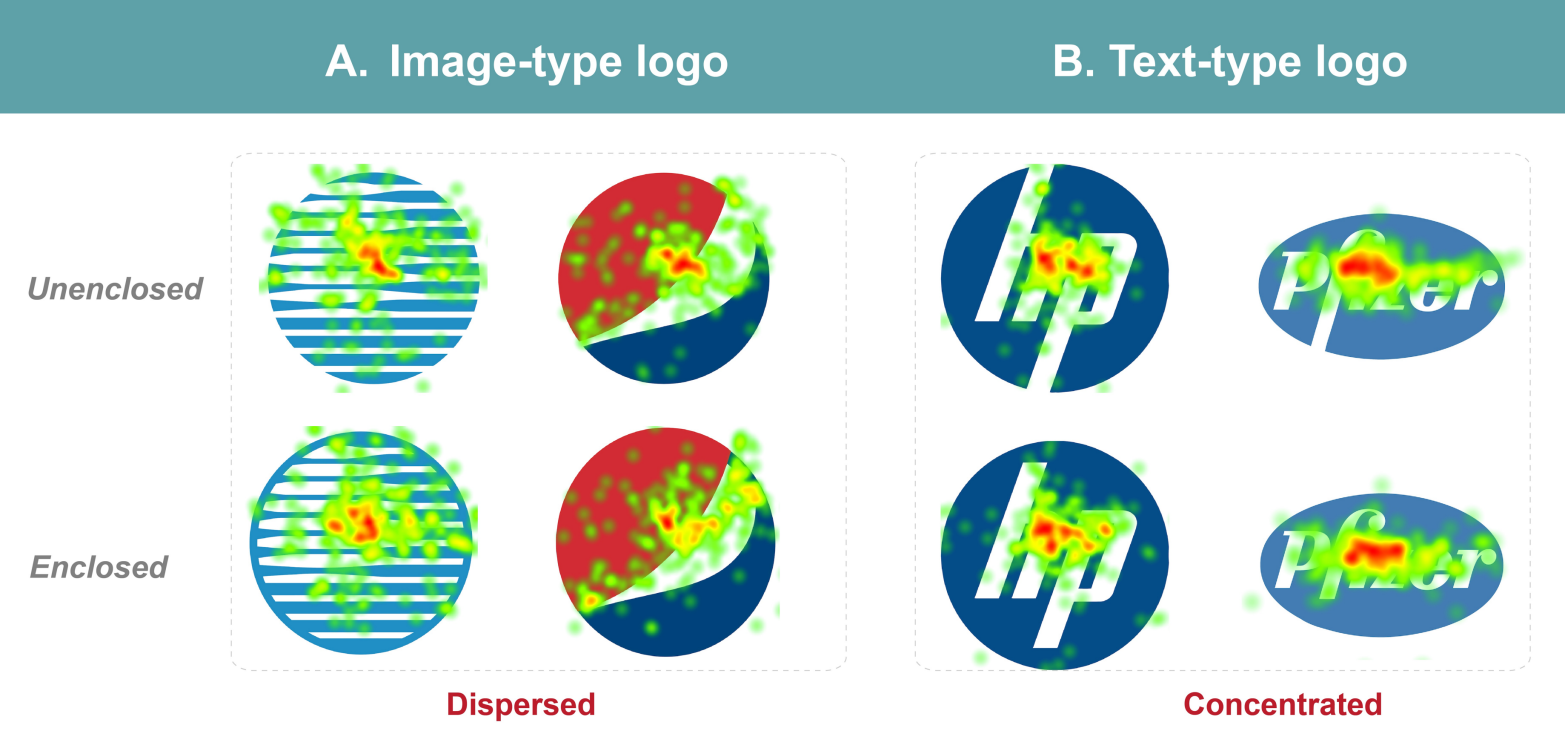
The heat map of different logo types

Further analysis of logotypes reveals that the distribution of fixation is significantly different (as shown in Table 2). Due to changes in cognition, Image-type logos exhibited the highest SDI value, more dispersed fixation points, and the most extended total fixation duration. The distribution of fixation was more concentrated for text-type logos (as shown in Figure 8). The control group contained newly designed logos that had never been seen by the public before. This group exhibited the shortest total fixation duration and had the highest complicacy and least aesthetic scores, which implies that the visual psychology for logos of all types may be influenced by familiarity. Michailidou (70) and Michailidou et al., (71) also pointed out that different types of logos exhibit different subjective complicacy and cognitive load.

**Table 2.**
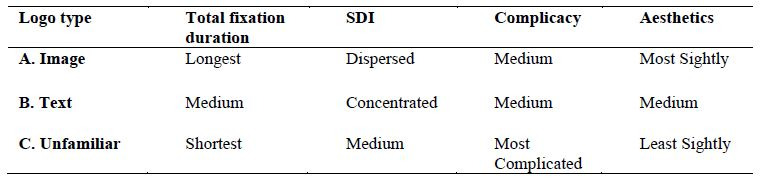
Comprehensive Analysis of Logo Types in Eye Movement Data and Subjective

### Enclosed and Unenclosed Logos and Their Subjective Psychological Evaluation

**Figure 9.**
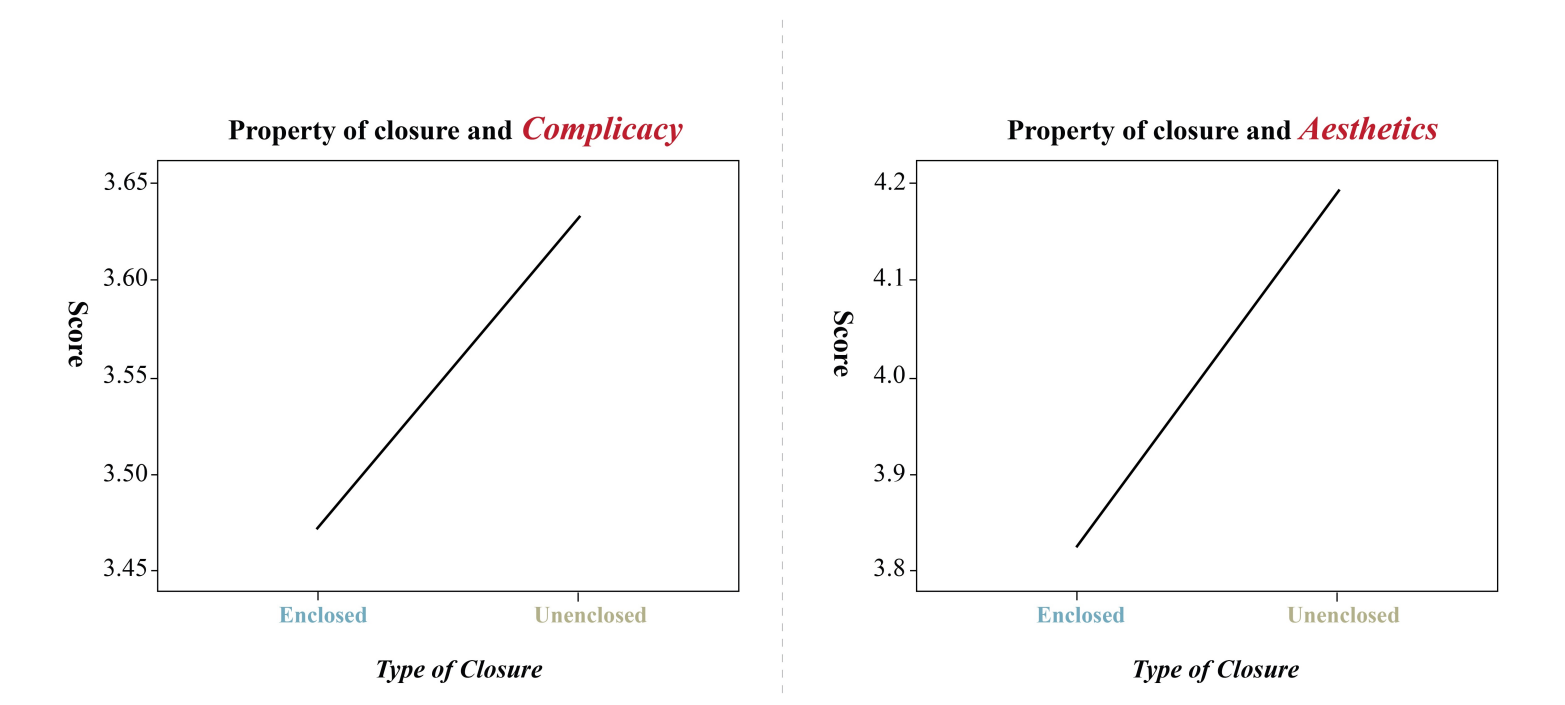
Subjective evaluation of enclosed logos

**Figure 10.**
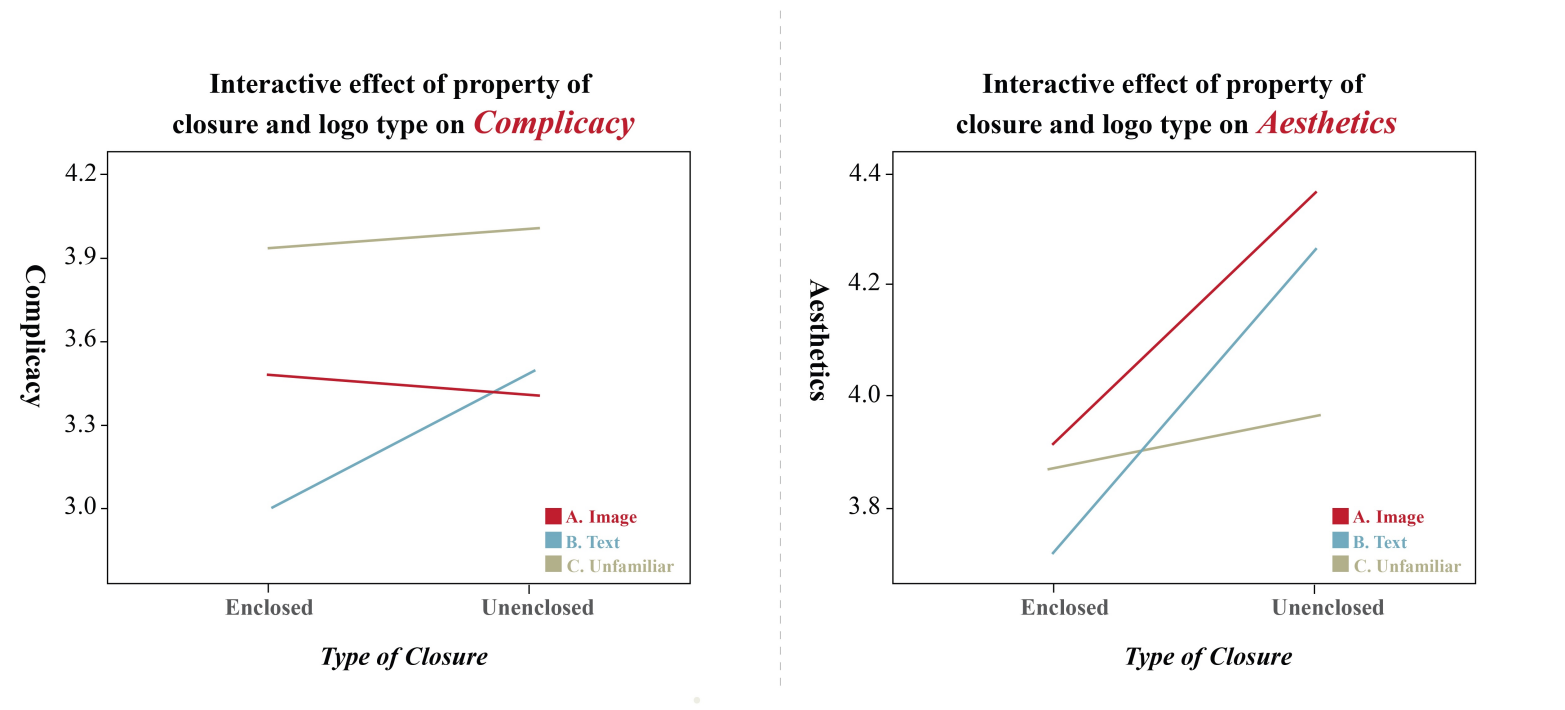
Properties of closure and logo type

The presence or absence of enclosure on the image had a significant main effect (*F*(1, 20) = 8.155, *p* < 0.01) on the complicacy-dependent variable and was also significantly affected by image type (*F*(2, 40) = 10.218, *p* < 0.01) There is a significant interaction between the two (*F*(2, 40) = 9.672, *p*<0.01), and the degree of complexity for enclosed images is different, indicating that enclosed logos have lower complicacy than non-enclosed logos, especially for text-type logos which have significantly lower complicacy than image-type logos and unfamiliar enclosed logos. The presence or absence of enclosure had a significant main effect (*F*(1, 20) = 7.452, *p* < 0.05) on the aesthetics-dependent variable and was insignificantly affected by image type (*F*(2, 40) = 1.125, *p*>0.05). Significant interaction exists between the two (*F*(2, 40) = 6.305, *p*<0.01), and conformity to the principle of enclosure have significantly different levels of aesthetics score, indicating that unenclosed logos are better looking than enclosed logos, as shown in Figure 9, Particularly, image-type logos are significantly more aesthetically pleasing than text-type logos and experimental unfamiliar non-enclosed logos, as shown in Figure 10.

## Discussion

### Principle of closure: The importance of the imaginary outline

This study analyzed design methodologies from the perspectives of empirical aesthetics and visual perception principles in Gestalt psychology. The data from the eye movement information and subjective evaluation revealed that whether or not a logo conformed to the principle of enclosure does not affect sightline significantly. According to the principle of closure, for an unenclosed logos, the viewer will automatically fill in the gaps during visual perception and regard a series of line segments as a complete line to form a complete image (76) (as shown in Figure 3), so the sightline information between the experimental group and the control group will be very similar. This study successfully confirmed the unseen yet crucial lines as dictated by the principle of closure by revealing that the unenclosed logos are recognized as complete images in addition to significantly improving the visual aesthetics. This research hypothesis 1 assumes that a completely closed logo and an open logo that conforms to the law of closure will automatically fill in the gaps as a property of the principle of closure, and there will be no significant difference in eye movement information. Research hypothesis 1 is established. The primary finding of this study is that the properties of the principle of closure are present in logo design, Chinese characters (66), outdoor advertising (76) as well as photography (24).

In terms of the subjective psychological evaluation and the principle of closure, Due to the decreased number of solid contours in the unenclosed logos, the features on the logo take more time to comprehend and cognitively connect into a complete shape, making evaluation more complex and in line with research findings from Burlinson et al., 2018; Elder and Zucker 1993, 1994; Kovács and Julesz, 1993; Li and Xuan, 2022. It was found that, between enclosed shapes and unenclosed shapes, the enclosed shapes have good recognizability and shorter visual search duration. It happens early in the visual process, indicating that the features of unenclosed and enclosed shapes do affect perceptual processing and the cognitive load of processing information is reduced (17; 18; 21). However, unenclosed logos align with psychological and physiological needs for aesthetics and comfort. Gestalt psychology also advocated that the goodness of perceptual stimulation depends on the relationship between the structure of the stimulant and the psychological functions. A 'good' form is easy to process and is essential in the generation of feelings of pleasure and aesthetic stimulation (6; 39; 56; 90).

**Figure 11.**
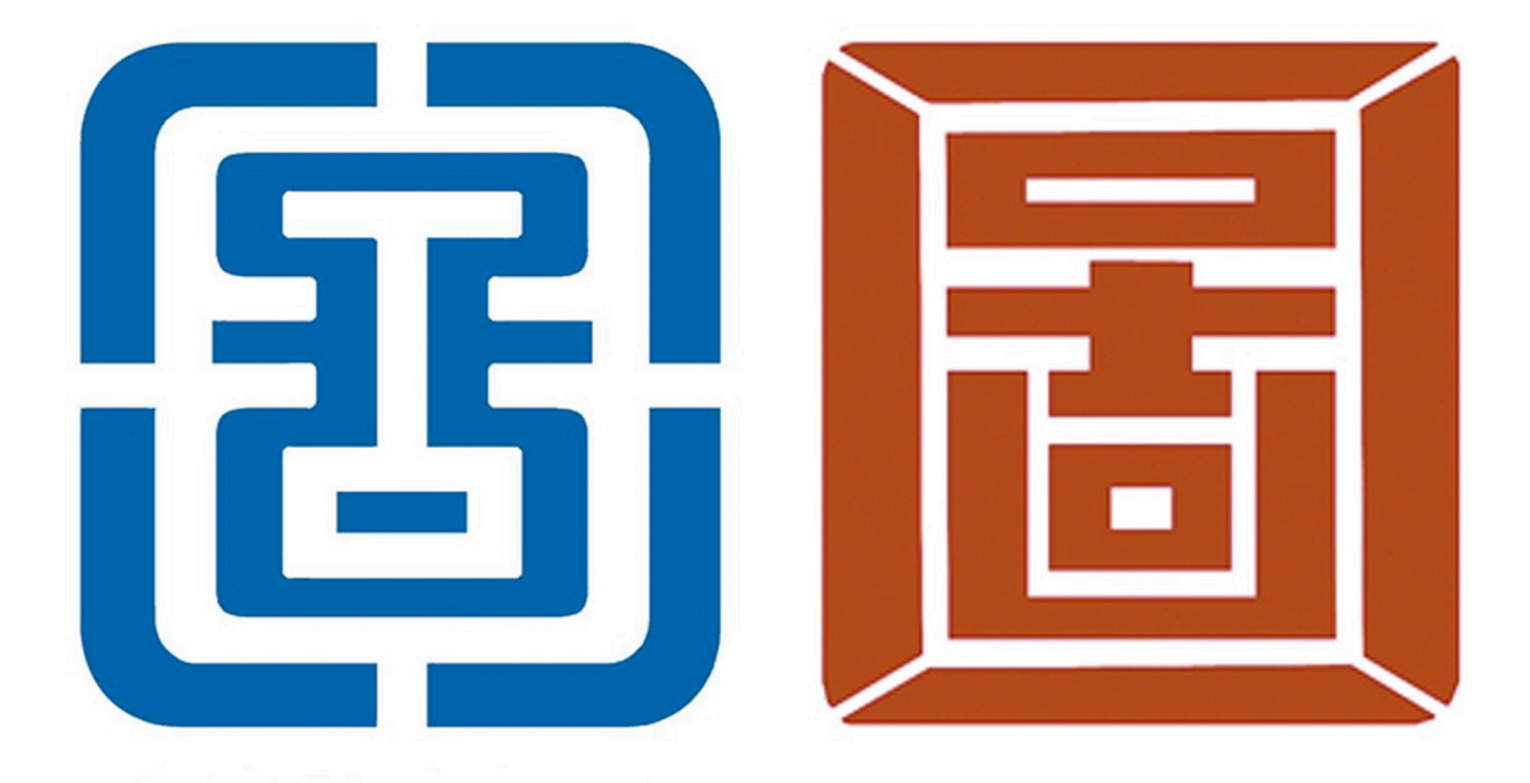
The open visual rhythm of enclosed character

The principle of closure is often used in logo design. It is a standard design method to leave intentional gaps. When the designer understands that the way people look at the logo is affected by the visual psychology and evaluation of the visual characteristics of the property of closure, they will begin to be aware of virtual unseen curves during their design process. Using the principle of closure to produce deliberately incomplete or imperfect graphics by virtualization or simplification, although subtracting part of the shape will make the logo look incomplete and closed, the viewer can automatically fill in the gaps into complete forms. This breaking of the rigidity of the logo has the potential to give the design new meaning and visual effects, creating a sense of novelty and visual beauty. This leaves more room for imagination for the viewer and makes the logo more eye-catching and intriguing, which can satisfy people's physical and psychological needs by generating feelings of simplicity, relaxation, freedom, and comfort and also prevent a sense of visual claustrophobia or aesthetic fatigue caused by complete closure. (26; 50; 60; 62; 75; 79; 90; 97), As Figure 11 illustrates, unenclosed characters exhibit the property of closure. Hypothesis 2 of this study: Logos that comply with the principle of closure have a high subjective psychological sense of aesthetics, particularly with logos that are partially enclosed, which produces a higher sense of comfort and pleasantness. Completely closed logos are less complicated but, at the same time, uninteresting and dull, which is in line with research Hypothesis 2.

**Figure 12.**
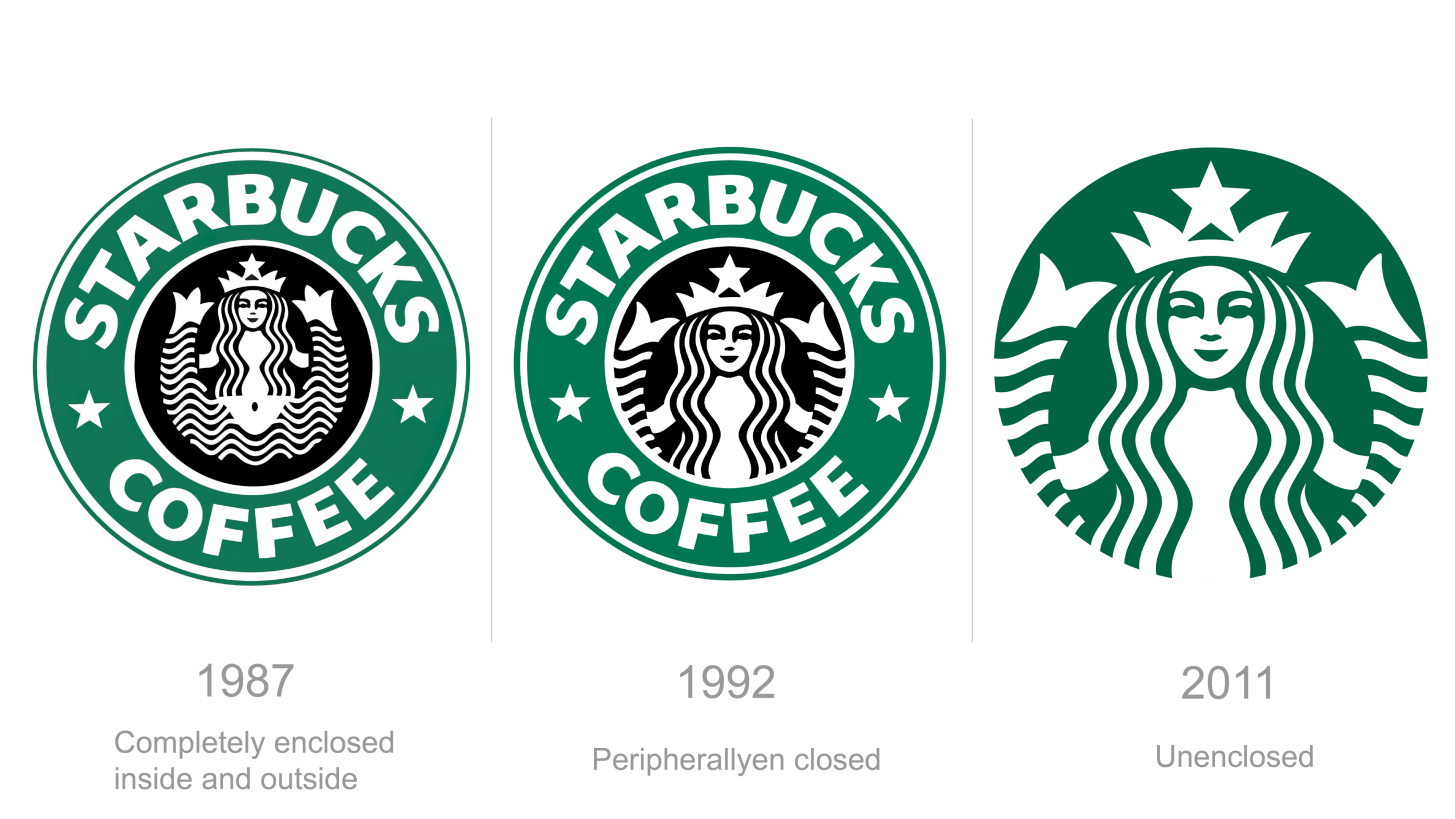
The evolution of Starbucks logo design, redesigned from being completely enclosed to an open logo conforming with the principle of closure

Chen and Bei (21) pointed out that framing plays a vital role in aesthetic creation, and this framing feature is widely used in art-related practices, especially the logo frame in design is a common brand design element but lacking in research (16; 33). As shown in Figure 12, As to the application of the principle of closure, the new Starbucks logo is an obvious example of applying findings from this research. It evolved from a completely enclosed shape to a simplified version that removed excessive unnecessary details, forming an unenclosed logo that conforms to the principle of closure that instills the brand with a sense of flexibility to explore product innovation (21; 93). The same is true for IBM's logo. The intentionally unenclosed design mitigates monotonicity and changelessness, and the use of Gestalt simplification does not destroy the integrity of the image. Not only does this allow the viewer to complete the logo recognition, but it also enhances the recognition with feelings of innovation and novelty, strengthening the unconscious logo registration. The comparison is innovative and exciting; the signs are recorded unconsciously (42). The current logos for Kia automobiles and Nokia both employ this design method.

### Conclusion

This study found that due to the principle of closure, completely closed and partially enclosed logos do not influence visual behavior; however, in subjective psychological feelings, concealed contour logos are more novel, interesting, and pleasant, which is in line with modern logo design trends. In addition, it was also found that image-type logos produce more dispersed sightlines and the longest fixation durations compared to text-type logos, which have more focused sightline distribution. However, it should be noted that too many missing parts in the graphics can affect recognition, so designers must master the balance between aesthetics and recognition (94). This research advocates that Real-world design applications must consider brand marketing strategy and orientation towards practicality or aesthetics. An image-type logo design that invokes closure is visually most sightly and is suitable for sensual advertisements and posters. On the other hand, with traffic signs and other types of practical signage, text-type logos that are entirely enclosed are recommended for the most effective recognition.

This study uses the quantitative research method of cognitive science to push forward an understanding of the application of the principle of closure in logo design and perceptual communication with the aid of eye movement experiments and subjective evaluations and found that the effects of the principle of closure in both completely enclosed and partially enclosed logos. Few researchers try integrating the two fields of art and cognition, making relevant research results quite rare. Designers who master the closure principle in Gestalt psychology, understand the basis of people's cognition and sense of aesthetics and make good use of the principle of closure in logo design can communicate visually more effectively by producing grabbing visual foci that make designs not just easier to identify and understand but also creating exciting novelty, However, differences in history and culture, language, customs, can influence emotions, thoughts, and aesthetic judgment differently in symbol design. This research can provide references and applications to teaching visual communication design practice, developing modern aesthetic theory, and strengthening artistic practice.

### Ethics and Conflict of Interest

The author(s) declare(s) that the contents of the article are in agreement with the ethics described in http://biblio.unibe.ch/portale/elibrary/BOP/jemr/ethics.html and that there is no conflict of interest regarding the publication of this paper.

### Acknowledgement

The authors acknowledge the eye movement analysis data Dr. Da-Lun Tang of Tamkang University provided.
